# A New Model of Acute Exacerbation of Experimental Pulmonary Fibrosis in Mice

**DOI:** 10.3390/cells11213379

**Published:** 2022-10-26

**Authors:** Céline-Hivda Yegen, Liasmine Haine, Kevin Da Costa Ferreira, Dominique Marchant, Jean-Francois Bernaudin, Carole Planès, Nicolas Voituron, Emilie Boncoeur

**Affiliations:** 1 Laboratoire Hypoxie & Poumon, UMR INSERM U1272, Université Sorbonne Paris-Nord, 93000 Bobigny, France; 2 Faculté de Médecine, Sorbonne Université, 75006 Paris, France; 3 Service de Physiologie et d’Explorations Fonctionnelles, Hôpital Avicenne, APHP, 93000 Bobigny, France; 4 Département STAPS, Université Sorbonne Paris-Nord, 93000 Bobigny, France

**Keywords:** animal model, lung fibrosis, inflammation, acute exacerbation

## Abstract

Rationale: idiopathic pulmonary fibrosis (IPF) is the most severe form of fibrosing interstitial lung disease, characterized by progressive respiratory failure leading to death. IPF’s natural history is heterogeneous, and its progression unpredictable. Most patients develop a progressive decline of respiratory function over years; some remain stable, but others present a fast-respiratory deterioration without identifiable cause, classified as acute exacerbation (AE). Objectives: to develop and characterize an experimental mice model of lung fibrosis AE, mimicking IPF-AE at the functional, histopathological, cellular and molecular levels. Methods: we established in C57BL/6 male mice a chronic pulmonary fibrosis using a repetitive low-dose bleomycin (BLM) intratracheal (IT) instillation regimen (four instillations of BLM every 2 weeks), followed by two IT instillations of a simple or double-dose BLM challenge to induce AE. Clinical follow-up and histological and molecular analyses were done for fibrotic and inflammatory lung remodeling analysis. Measurements and main results: as compared with a low-dose BLM regimen, this AE model induced a late burst of animal mortality, worsened lung fibrosis and remodeling, and superadded histopathological features as observed in humans IPF-AE. This was associated with stronger inflammation, increased macrophage infiltration of lung tissue and increased levels of pro-inflammatory cytokines in lung homogenates. Finally, it induced in the remodeled lung a diffuse expression of hypoxia-inducible factor 1α, a hallmark of tissular hypoxia response and a major player in the progression of IPF. Conclusion: this new model is a promising model of AE in chronic pulmonary fibrosis that could be relevant to mimic IPF-AE in preclinical trials.

## 1. Introduction

Idiopathic pulmonary fibrosis (IPF) is the most severe form of chronic fibrosing interstitial lung disease (ILD), histologically defined by a usual interstitial pneumonia (UIP) pattern. IPF is thought to be the consequence of repetitive micro-injuries of alveolar epithelial cells (AEC), followed by inefficient epithelial repair and uncontrolled activation and proliferation of interstitial (myo) fibroblasts [1]. Mainly observed in the elderly, it leads to a progressive and fatal respiratory failure with a mean survival of 3–5 years from the time of diagnosis [2]. There is no curative medical treatment for IPF except lung transplantation when feasible, and current antifibrotic drugs only slow down the decline of respiratory function, urging researchers to identify new therapeutic strategies. IPF’s natural history is heterogeneous, and its evolution unpredictable. While most patients present a progressive decline of respiratory function over years, some patients remain stable, and others may present a fast deterioration of pulmonary function and respiratory failure without any identifiable cause such as volume overload or heart failure [3,4]. This rapid respiratory deterioration has been classified as acute exacerbation (AE) of IPF (IPF-AE). Clinically, IPF-AE is defined by acute worsening or development of dyspnea occurring in typically less than one month. Computed tomography reveals new bilateral ground-glass opacities or airspace consolidation superimposed on the underlying UIP pattern. On histological biopsies, IPF-AE is characterized by a combination of a UIP pattern associated with superimposed acute lung injury (ALI) with diffuse alveolar damage and organizing pneumonia [5]. The occurrence of IPF-AE cannot be predicted or prevented, and it has a bad prognostic impact in the absence of efficient medical treatment, with an in-hospital mortality of about 50% because of respiratory failure, and an 80% mortality at one year [3]. The molecular mechanisms underlying the ALI observed during these exacerbation phases are still poorly known and need further investigation.

To understand the physiological and molecular mechanisms involved in the development and progression of IPF, murine models of pulmonary fibrosis have been developed. The most widely used murine model consists of a single intra-tracheal (IT) instillation of bleomycin (BLM) at a dose ranging from 2 IU/g to 3.5 IU/g. Following strong lung inflammation during the first week post-instillation, lung fibrosis develops within 2–3 weeks, but with a very heterogeneous distribution of lesions, which does not realistically replicate the UIP histological features [6]. Moreover, a spontaneous resorption of fibrosis is observed in surviving mice, which does not reproduce the natural progressive course of IPF [7,8,9]. More recently, a major improvement has been brought in these BLM mouse models by using repetitive administration of low-dose BLM (3 to 8) IT instillations of 0.8 UI/g BLM spaced two weeks apart), based on the IPF pathogenic hypothesis of repetitive alveolar micro-injuries. These models, although not yet fully characterized at the cellular/molecular level, have the advantage of inducing a persistent lung fibrosis without major inflammation, reproducing histology features observed in UIP [10,11]. 

A suitable preclinical model of ALI occurring on a chronic pulmonary fibrosis background to mimic IPF-AE is urgently needed since IPF-AE is a major event in the course of IPF. To our knowledge, reports dealing with AE models superadded on lung fibrosis are scarce. These models include AE induced by gamma herpes virus infection in mice [12,13,14], and only one noninfectious model has been proposed [15]. Therefore, the goal of the present study was to develop and characterize a novel mouse model of non-infectious AE occurring in a chronically fibrotic lung and mimicking IPF-AE. To do so, we first established a chronic pulmonary fibrosis using a repetitive low-dose BLM regimen (four IT instillations of BLM 0.8 UI/g spaced two weeks apart), and induced AE by two additional IT instillations of a double-dose BLM challenge (1.6 UI/g). The characterization of this model at the functional, histopathological, cellular and molecular levels indicates that it fairly mimics IPF-AE and that it is a promising tool for future preclinical trials. 

## 2. Materials and Methods

### 2.1. Ethical Approval

Mouse models and experimental procedures were authorized by the French Ministry of Higher Education, Research and Innovation (APAFIS #18309-2019010316127879 v16) and done in accordance with the European Community’s council directive 2010/63/EU for animal care.

### 2.2. Animals

Experiments were performed on 8-week-old C57BL/6 male mice weighing an average of 25 g ± 2 g (Janvier Labs, Le Genest-Saint-Isle, France). The animals were acclimatized for at least 5 days to the local animal facility and housed in standard conditions in a 12 h/12 h light/dark cycle, at an ambient temperature of 20–22 °C and had ad libitum access to water and food.

### 2.3. Experimental Design

**Figure 1:**
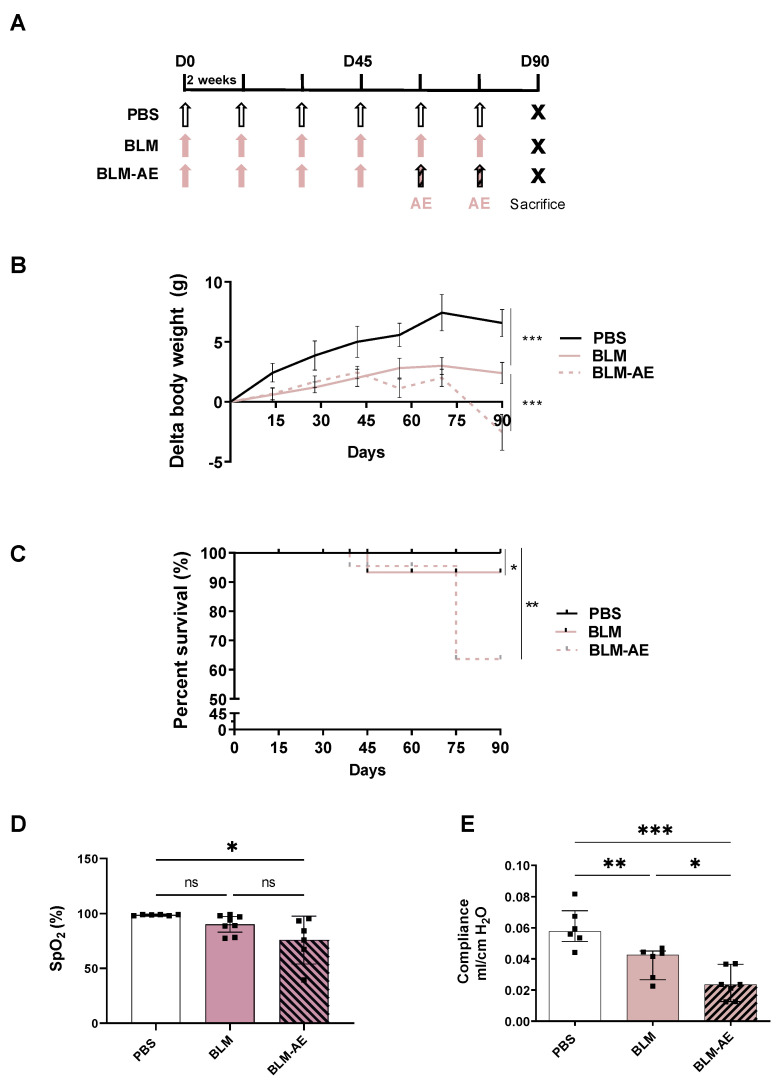
Experimental protocol, survival and weight curves. (**A**) Eight-week-old C57BL6/J male mice received six IT instillations (arrows) of bleomycin (BLM) at 0.8 IU/g or PBS every two weeks to form the BLM (pink arrow) and PBS groups (white arrow), respectively. In parallel, a group of mice received four IT instillations of 0.8 UI/g BLM, and then two IT instillations of BLM at 1.6 UI/g, thus forming the acute exacerbation group (BLM-AE, pink-hatched black arrow). Mice were sacrificed at day 90 (D90) for analyses. (**B**) Body-weight monitoring was established throughout the experiment, the difference in weight compared to the weight measured on day 0 (D0) before the IT instillations of PBS (n = 6, black line), BLM, (n = 6, dotted pink line) and BLM-AE (n = 7, pink line) groups are reported on a graph. A Friedman test followed by a Dunn’s multiple comparisons test was performed to estimate the difference in mice weight as compared to initial weight, *** *p* < 0.001. (**C**) Mouse survival was recorded every day until the end of the experimental design for the different groups (PBS, black line; BLM, dotted pink line; and BLM-AE, pink line) and plotted on a Kaplan–Meier curve. (**D**) Mouse peripheral oxygen saturation (SpO2) was measured by infrared pulse oximetry. (**E**) Lung compliance was measured by plethysmography. (**D**,**E**) Data were presented as a box plot representing the median ± interquartile range. Raw data were submitted to one-way ANOVA test followed by Newman–Keuls to compare each group (control PBS group (white bar), BLM group (pink bar) and BLM-AE group (pink hatched black bar)). ns: not significant, * *p* < 0.05, ** *p* < 0.01, *** *p* < 0.001, respectively.

Lung fibrosis was induced by intra-tracheal (IT) instillations of 0.8 UI/g body weight bleomycin (BLM) (Sigma-Merck, Saint-Quentin-Fallavier, France), in 100 μL phosphate buffer saline (PBS) every two weeks under 4% isoflurane anesthesia as already described [16]. Briefly, an intubation stand with adjustable head positioning (Kent Scientific) was used to fixate the mice during the intubation procedure while anesthesia was maintained. The mice were placed on the plastic support with the nose positioned in a facemask. The mice were suspended by the upper teeth and the neck was placed with an angle of 45° backward. A fiberoptic from an external halogen light source was placed at the front neck of the mice to illuminate the larynx and make the vocal cords visible. Then, a homemade laryngoscope (a syringe connected to a 26G catheter) was used. It was inserted in the direction of the larynx under the control of a binocular loupe and the catheter was pushed into the trachea, passing between the vocal cords. The instillation is carried out only at this moment. The mice were monitored in cages until they had fully recovered. Mice were weighed daily. At the beginning of the experiments, 31 mice were randomly divided into 3 groups: 8 mice receiving 6 instillations of Phosphate Buffer Saline (PBS group), 10 mice receiving 6 instillations of 0.8 UI/g body weight BLM in 100 μL PBS (BLM group) and 13 mice receiving 4 instillations of 0.8 UI/g body weight in 100 μL PBS and for the last 2 instillations 1.6UI/g body weight BLM in 100 μL PBS to induce acute exacerbation (BLM-AE group) (Figure 1A). At the end of the experiments, due to BLM-induced mortality, 25 mice were alive (8 mice for the PBS group, 9 mice for the BLM group and 8 mice for the BLM-AE group). 

Two weeks after the last instillation (D90), mice were sedated by intraperitoneal injection of a mixture of ketamine 1000 (Virbac, 06510 Carros, France) and xylazine rompen 2% (Bayer Healthcare, Gaillard, France) (ketamine/xylazine; 100 mg/kg and 20 mg/kg respectively), tracheotomized and ventilated for the measurement of compliance or exsanguinated before lung sample collection (Figure 1A).

*Lung compliance measurement by plethysmography:* Pulmonary compliance was evaluated on six mice from the PBS group, six mice from the BLM group and seven mice from the BLM-AE groups using a plethysmograph (Emka technologies, Paris, France) as previously described [17]. Briefly, the mice were ventilated (RoVent Jr., Kent Scientific Corporation; respiratory frequency = 150 bpm, tidal volume = 0.27 mL; Insp/Exp ratio = 0.40). A differential pressure transducer was used to obtain a flow signal that reflects the expansion and contraction of the thorax during each ventilation cycle. Compliance and resistance were calculated following flow signal and pressure signals acquisition. Volume signal was obtained by an integration of the flow signal measured by the differential pressure transducer. The pneumotachograph being the only way for air to flow into and out of the chamber, the difference of pressure between inside and outside was proportional to this flow.

*Oxygen saturation (SpO2) monitoring:* Oxygen saturation was assessed by a non-invasive method of infrared pulse oximetry (mouseOx Plus), as previously described [18], two weeks after the last instillation. 

### 2.4. Lung Sampling for Analyses

Lung samples were obtained as previously described [16]. At the end of the experiment, the surviving 25 mice were tracheotomyzed and exsanguinated; PBS was injected into the right ventricle of the heart to rinse the lung vasculature. The right lobe of the lung was isolated through a ligation to prevent the passage of instillated products. The left lobe was inflated and fixed with 4% paraformaldehyde at a pressure of 20 cm H_2_O through the tracheostomy cannula. The heart and lungs were removed “en bloc”. The right lung was split into three samples (one per lobe) and placed in liquid nitrogen and stored at −80 °C for molecular analyses (RNA and proteins). The left lung was placed in 20 mL of 4% PFA for 24 h, paraffin embedded and 5 µm sections were cut.

### 2.5. Microscopy Analyses

*Tissue staining:* Hematoxylin-eosin staining: 5 µm sections from the left lung of six mice from the PBS group, six mice from the BLM group and seven mice from the BLM-AE groups were stained using Harris hematoxylin (Sigmaaldricht, MHS16, Saint-Quentin-Fallavier, France) and aqueous eosin solution (Merck, Art.1144, Saint-Quentin-Fallavier, France). The extent of lung injury was estimated by quantification of tissue optic density using HistoLab® Image Analysis Software version 10.1 (Microvision Instrument, Evry, France). The normally aerated alveolar spaces area was calculated, as well as the total area of the section, the injured area was reported as the percentage of the total lung section. Masson’s trichrome staining: Additional 5 µm sections of the same groups of mice were incubated with phosphomolybdic/phosphotungstic acid solution (5 min), stained 5 min with aniline blue solution (VWR International, Rosny-sous-Bois, France), and then rinsed with 1% acetic acid solution (2 min). Sirius red/fast green staining: Additional 5 µm sections of the same groups of mice were stained using 0.1% fast green (Merck, F7252, Saint-Quentin-Fallavier, France) and 0.1% direct red 80 (Sigmaaldricht, Cat#365548, Saint-Quentin-Fallavier, France). 

*Immunohistochemistry:* IHC was performed on tissue sections from six mice from the PBS group, six mice from the BLM group and seven mice from the BLM-AE. Antigen retrieval was performed in a boiling citrate buffer (10 mM sodium citrate, 0.05% Tween 20, pH 6.0). Endogenous peroxidases were quenched with 3% hydrogen peroxide for 10 min and sections were incubated with 5% of normal horse serum for 1 h to block nonspecific antibody binding sites. Sections were incubated with the different primary antibodies overnight at 4 °C (for detail see Table 1). The next day, slides were incubated with biotin-conjugated secondary antibodies (Dako REAL ™ Detection System, Peroxidase/DAB+, Rabbit/Mouse, Les Ulis, France) for 10 min and then with peroxidase-bound streptavidin (HRP) for 10 min. DAB (3,3’-diaminobenzidine) solution and nuclear fast red (Sigma Aldrich, ref 60700, Saint-Quentin-Fallavier, France) staining were used to visualize the positive reactions. 

*Inflammatory cell quantification:* Random fields of stained lung biopsies from six mice from the PBS group, six mice from the BLM group and seven mice from the BLM-AE were photographed. Quantification has been done on the whole left lung. For each field in each mouse and in each condition, the average number of positive cells for F4-80, CD3 and CD19 immunolabelling was reported to the total number of cells present.

### 2.6. Biochemical and Molecular Biology Analyses

*Soluble lung collagen:* The amount of soluble pulmonary collagen, comprising the collagen released after enzymatic digestion with 0.1 mg/mL pepsin (516360-500MG, EMD Millipore, MERK, Saint-Quentin-Fallavier, France), was measured on the right superior lobe of six mice from the PBS group, six mice from the BLM group and seven mice from the BLM-AE groups using the Sircol test (Biocolor Ltd., Belfast, UK) according to the manufacturer’s instructions.

*ELISA:* Inflammatory markers were done on six mice from the PBS group, six mice from the BLM group and seven mice from the BLM-AE groups. Interleukin (IL-6, CXCL1/KC, IL1β) and tumor necrosis factor alpha (TNF-α) concentration were assessed using 50 μg of total protein extracts according to the manufacturer’s instructions (Mouse IL-6 DuoSet ELISA DY406-05, Mouse CXCL1/KC DuoSet ELISA DY453-05, Mouse IL1β Duoset ELISA DY401-05 and Mouse TNF-αDuoSet ELISA MTA00B, R&D System, biotechne, Noyal Châtillon sur Seiche, France). The absorbance was measured at 450 nm with a BIO-RAD, Model 680 Microplate Reader.

*Reverse transcription–quantitative polymerase chain reaction:* 30 mg of lung tissue were placed in 800 μL of TRIzol reagent (Qiazol® Lysis reagent, Courtaboeuf, France) and homogenized on ice using an Ultra-Turrax homogenizer. RNA extraction was performed following the manufacturer’s instructions. Total RNA was quantified by measuring the absorbance at 260 nm, using a Nanodrop reader (Shimdazu, BioSpecNano software, Marne-la-Vallée, France). Extraction quality and the purity of the RNA (260 nm) were evaluated using 260 nm/280 nm and 260 nm/230 nm absorbance ratios. Single-strand cDNAs were synthesized from 0.5 μg of total RNA according to the manufacturer instructions (Maxima First Strand cDNA Synthesis Kit Reaction Mix, ThermoFisher scientific, Ilkirch, France). 

Resulting cDNA samples were 1:10 diluted and amplified by PCR conducted with absolute qPCR SYBR green ROX mix (Fisher Scientific, Illkirch, France) on StepOne system qPCR (Applied Biosystems, Life Technologies, France). Cycle threshold values were normalized to amplification of the ribosomal 18S sub-unit. Relative quantification was performed using the 2^−ΔΔCt^ method (AB Applied Bio Systems, Step One Plus Real Time PCR) on six mice from the PBS group, five mice from the BLM group and five mice from the BLM-AE groups. The primer sequences used for quantitative real-time PCR are listed in Table 2. 

### 2.7. Statistics

Graph and statistical analyses were performed using GraphPad Prism (GraphPad Software, version 9). Data are presented as a box plot representing the median ± interquartile range. Shapiro–Wilk and Kolmogorov–Smirnov tests demonstrate a normal distribution of the data. Comparisons among groups were assessed using ordinary one-way ANOVA followed by post hoc Newman–Keuls tests. *p* < 0.05 differences were considered significant.

## 3. Results

### 3.1. Acute Injury by Last Double-Dose Bleomycin Induces a Rapid Clinical and Respiratory Deterioration

In the BLM and BLM-AE groups, a significant defect in the expected physiological weight gain was observed next to D15 (Figure 1B). After the fourth injection at D45, the body weight was stabilized in the two BLM and BLM-AE groups, while a significant decrease in body weight was observed after D70 only in the BLM-AE mice. The mortality observed in the BLM group was limited (<10%), with animal death occurring around day 45 (D45), but not afterward (Figure 1C). Mortality increased dramatically in the BLM-AE mice at D75 (reaching approximately 40% at D90), whereas no death was noted in the BLM group during the same period. At the end of the experiments (D90), a significant decrease in SpO2 was observed in the surviving mice of the BLM-AE group (but not in the BLM group), as compared to the PBS group (Figure 1D). In addition, lung compliance measured by plethysmography at D90 (Figure 1E) was reduced in both BLM and BLM-AE groups as compared to the PBS group, and the decrease was significantly greater in the BLM-AE group as compared to the BLM group.

### 3.2. Acute Injury by Last Double-Dose Bleomycin Instillation Increases Lung Remodeling and Worsens Fibrosis

**Figure 2:**
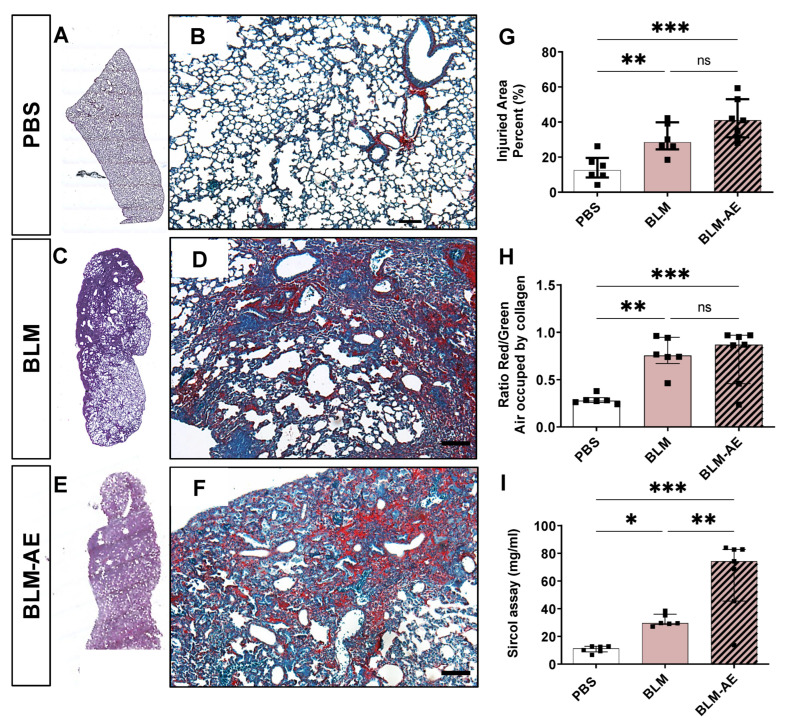
Histological study and analysis of collagen deposits. (**A**,**C**,**E**) Representative mapping of hematoxylin and eosin-stained from the PBS (**A**), BLM (**C**) and BLM-AE (**E**) groups to evaluate global lung architecture. (**B**,**D**,**F**) Representative Sirius red and fast green lung sections from the PBS (**B**), BLM (**D**) and BLM-AE (**F**) groups (scale bar corresponding to 100 µm) to evaluate collagen deposits and lung remodeling. (**G**) Lung injured area was estimated by quantification of sections density after H&E staining using HistoLab® image analysis software and expressed as the percentage of the total lung section. Injury quantification was normalized to the mean value of control group and presented as a percentage. (**H**) Light microscopy quantification of Sirius red staining reported to fast green staining quantification. (**I**) Quantification of soluble collagen in the right lung middle lobe by the Sircol method. (**G**–**I**) Data were presented as a box plot representing the median ± interquartile range. Raw data were submitted to one-way ANOVA test followed by Newman–Keuls to compare each group (control PBS group (white bar), BLM group (pink bar) and BLM-AE group (pink hatched black bar)), ns: not significant, * *p* < 0.05, ** *p* < 0.01, *** *p* < 0.001, respectively.

As shown by cartography of hematoxylin–eosin (HE)-stained lung sections, BLM treatment induced marked changes in lung morphology as compared to PBS (Figure 2A,C). Quantification of injured areas by HE staining (Figure 2G) confirmed significant injured lung areas in the BLM and BLM-AE groups as compared to the PBS group, and an increase (while still below the significance level) in the BLM-AE group as compared to the BLM group. 

Observation of sections stained by Sirius red/fast green (Figure 2B,D,F) showed an excessive extracellular matrix with compact collagenous deposits associated with air space reduction in BLM and BLM-AE lungs. The ratio of the red/green staining, which represents the proportion of collagen vs. cellular background, respectively, confirmed this observation (Figure 2H). Finally, quantification of lung total collagen content by the Sircol method revealed a significant burst in collagen synthesis in the BLM-AE group, with a collagen content in this group twofold higher than in the BLM group (Figure 2I). 

**Figure 3:**
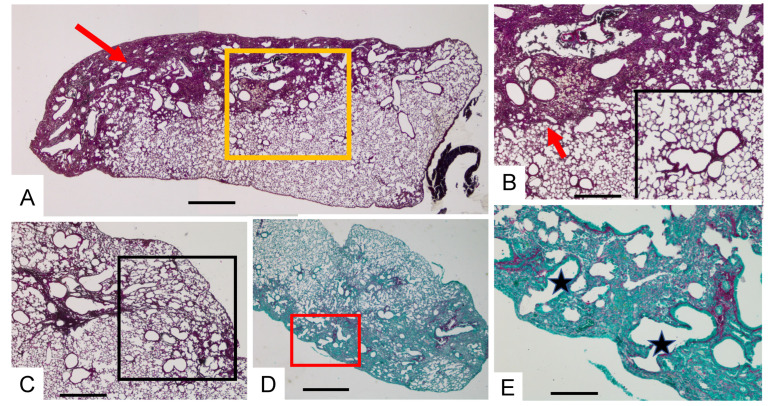
Lung histopathology in the BLM group. Illustration of lung lesions observed in different mice after repetitive low-dose BLM IT instillations. (**A**) Subpleural distribution with inward extension of the dense lung remodeling (red arrow) with cystic airways remnants (H&E bar 1500 µm). (**B**) Higher magnification of the A area framed in yellow showing a neat demarcation (red arrow) with the normal lung (scale bar 500 µm) magnified 4x in the insert. (**C**) Subpleural remodeling showing alveolar collapse especially in the framed area (H&E bar 500 µm). (**D**,**E**) Dense fibrotic areas with cystic airways reaching the pleural limit (stars). (**E**) is a magnification of the (**D**) framed area (Sirius red/green staining; (**D**): scale bar 1500 µm; (**E**): scale bar 300 µm).

When focusing the histopathology analysis on the BLM and BLM-AE groups, lung remodeling reminiscent of the UIP pattern was observed in both groups, with additional features in the BLM-AE group. In the lung of the BLM group, a dense remodeling made of fibrosis and cellular infiltrate was constantly observed in a subpleural distribution with various inward extension (Figure 3A,B). Remnants of airway lumen contribute to give a cystic pattern reminiscent of the honeycomb cysts observed in UIP in the subpleural tissue. A clear demarcation of the confluent fibrotic area with the preserved normal lung was a notable observation. Characteristic alveolar collapse was constantly observed (Figure 3C), but mostly encased in the fibrocellular changes. This pattern evocative of the one observed in UIP was reinforced by the presence of airway “traction” resulting in contact with pleura as observed in traction bronchiectasis in IPF patients (Figure 3D,E). 

**Figure 4:**
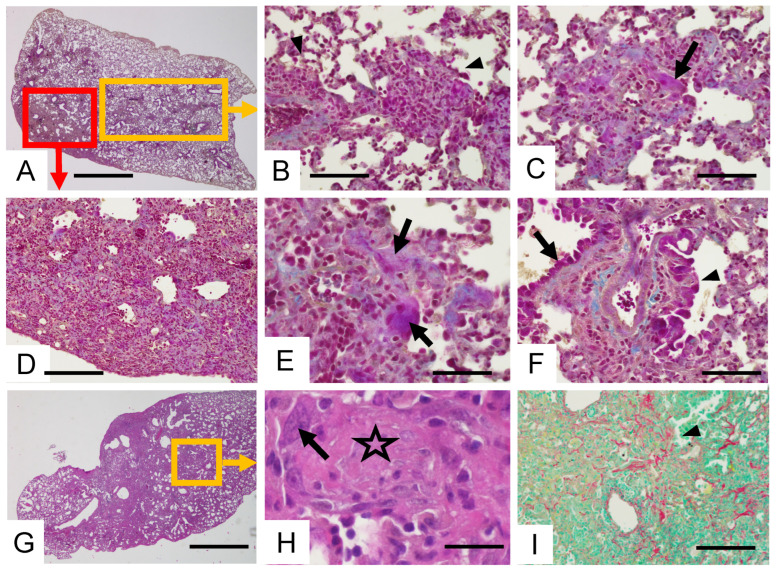
Lung histopathology in the BLM-AE groups. Illustration of lung lesions observed in different mice after double-dose BLM intratracheal instillations. (**A**) Subpleural dense cellular and fibrotic remodeling observed by Masson’s staining. Scale bar 2000 µm. (**D**) Magnification of the red framed area in A, scale bar 200 µm. (**B**,**C**,**E**,**F**) Magnification of the yellow framed area in A showing mononuclear cell infiltration. (**B**) Perivascular mononuclear cell infiltration (arrowheads). Scale bar 100 µm. (**C**,**E**) Acute alveolar cell lesions (arrows) and blue matrix deposits. (**C**) Scale bar 100 µm, (**E**) scale bar 150 µm, (**F**) “Reactive” airway cells (arrowhead) compare to normal cells (arrows). Scale bar 150 µm. (**G**–**I**) Subacute lesions in a dense remodeled area. (**G**) Masson’s staining. Scale bar 200 µm. (**H**) Magnification of the yellow framed area in G showing an interalveolar bud (star) layered by atypical epithelial cells (arrow). Scale bar 50 µm. (**I**) Red-stained intercellular collagen deposits (arrowhead) within the cellular infiltration (Sirius red/fast green staining, scale bar 100 µm).

In the lung of the BLM-AE group, lesions already described in the BLM group were observed but with superadded features as shown in Figure 2F and Figure 4A. First, the spared lung, which was distinctly delimited from the remolded area in BLM mice, showed multiple scattered, mostly perivascular mononuclear cell infiltrates (Figure 4B,C,E,F) interspersed with collagen fibers. In these lesions, subacute alveolar cell damages with matrix deposits were observed (Figure 4D,F,I). Organized intra-alveolar or interstitial loose collagenous fibromyxoid scars evocative of organizing pneumonia lesions were present, as illustrated by intra-alveolar buds layered by dysplastic alveolar epithelial cells (Figure 4G,H). In addition to alveolar cells, reactive airway epithelial cells were also observed (Figure 4F). 

### 3.3. Lung Inflammation Is Observed in the Acute Exacerbation Model of Pulmonary Fibrosis

**Figure 5:**
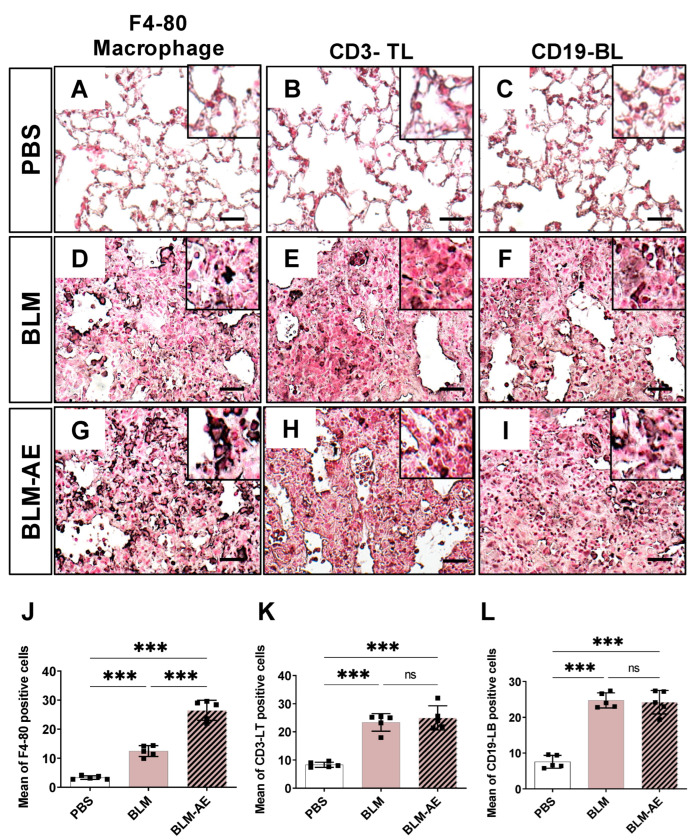
Identification of macrophage and lymphocyte cell populations. (**A**,**D**,**G**) Immunostained representative lung sections for F4-80 macrophages, (**B**,**E**,**H**) CD3 T lymphocytes and (**C**,**F**,**I**) CD19 B lymphocytes identification in the PBS (**A**–**C**), BLM (**D**–**F**), BLM-AE (**G**–**I**) groups. Scale bar corresponding to 100 µm. A representative image of the PBS, BLM and BLM-AE groups is shown. After BLM instillation, infiltration of F4-80 macrophages is observed in the BLM group (**D**) and an upsurge is shown after exacerbation (BLM-AE, (**G**)). T lymphocyte infiltration was observed in the BLM group ((**E**,**F**), respectively) and after exacerbation ((**H**,**I**), respectively). (**J**) Quantification of macrophages, (**K**) CD3-LT and (**L**) CD19-LB in the control PBS group (white bar), BLM group (pink bar) and BLM-AE group (pink hatched black bar). Six mice from the PBS group, six mice from the BLM group and seven mice from the BLM-AE were photographed. For each field in each mouse and in each condition, the average number of positive cells for F4-80, CD3 and CD19 immunolabelling was reported to the total number of cells present. All values are represented as median ± interquartile range; one-way ANOVA analysis followed by Newman–Keuls was performed. ns: not significant *** *p* ≤ 0.001.

Macrophages (Figure 5A,D,G) and T (Figure 5B,E,H) or B lymphocytes (Figure 5C,F,I) were present within the remodeled interstitium of the BLM or BLM-AE groups but not in the PBS group. F4-80 macrophages were observed in the fibrosis area in the BLM group or in the vicinity of the fibrosis (Figure 5D). A marked upsurge of these cells was observed after exacerbation (Figure 5G). T (Figure 5E) and B lymphocytes (Figure 5F) were present in the remodeled areas of both the BLM and BLM-AE groups. Quantification of inflammatory cells showed that exacerbation induced a doubling in the number of F4-80 macrophages (Figure 5J), while it did not significantly affect the number of CD3-LT (Figure 5K) or CD19-LB lymphocytes (Figure 5L), as compared to BLM.

**Figure 6:**
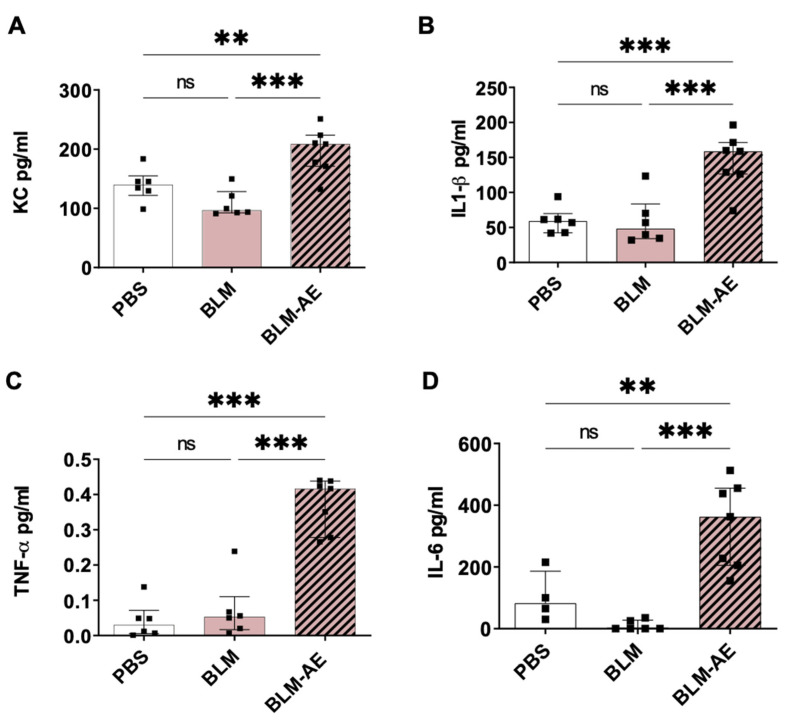
Quantification of pro-inflammatory cytokines in whole lung extracts: Quantification of KC (**A**), IL-1*β* (**B**), TNF-*α* (**C**) and IL-6 (**D**) pro-inflammatory proteins in whole-lung protein lysates from the control PBS group (white bar), BLM group (pink bar) and BLM-AE group (pink hatched black bar). All values are represented as median ± interquartile range; one-way ANOVA analysis followed by Newman–Keuls was performed. ns: not significant, ** *p* ≤ 0.005, *** *p* ≤ 0.001.

Lung inflammation was further quantified through the expression of pro-inflammatory cytokines in lung extracts. Levels of KC (Figure 6A), IL-1*β* (Figure 6B) TNF-α (Figure 6C) and IL-6 (Figure 6D) were not significantly modified in the BLM group as compared to the PBS group. Interestingly, a significant induction of KC (Figure 6A), IL1-*β* (Figure 6B), TNF-α (Figure 6C) and IL-6 (Figure 6D) was observed in the BLM-AE group as compared to the BLM and PBS groups. 

### 3.4. HIF-1a Is Associated with the Worsening Effect of Acute Injury on Lung Fibrosis

**Figure 7:**
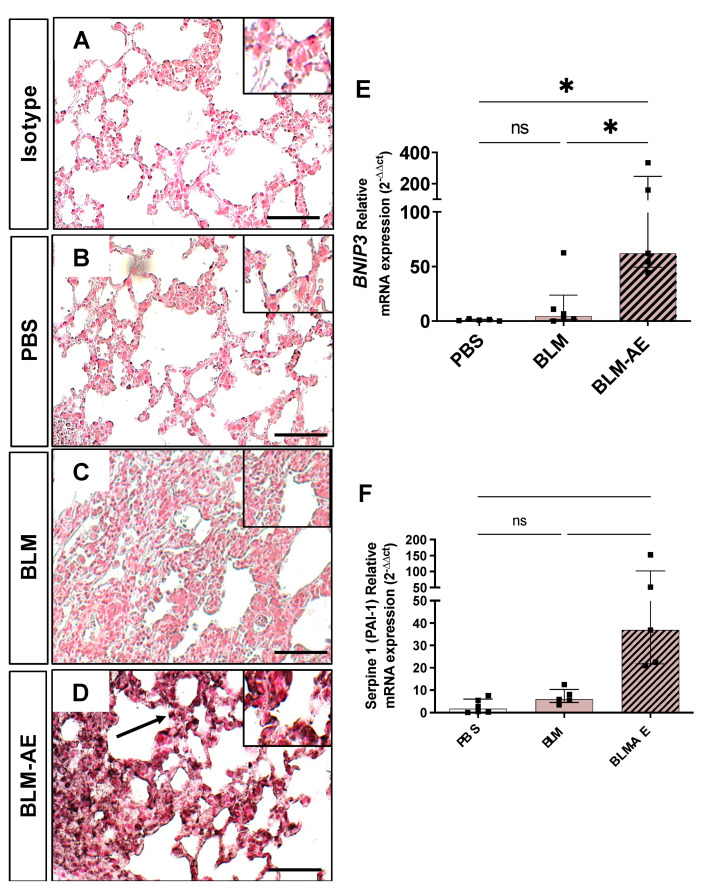
Hypoxia-induced factor HIF-1*α* immunostaining and expression level of target genes. (**B**–**E**) Photographs of 5 µm serial lung sections immunostained for HIF-1*α* protein and a rabbit isotype. (Scale bar corresponding to 50 µm). A representative image of the PBS (**B**), BLM (**C**), and BLM-AE (**D**) groups was shown. When reported to the staining of isotype (**A**), no significant HIF-1*α* staining was observed in the BLM group as compared to the PBS control group. Significant increase staining of HIF-1*α* is observed in the BLM-AE group mainly localized in modified alveolar epithelial cells (black arrow) (**D**). (**E**) mRNA expression of BNIP3 and (**F**) mRNA of SERPINE1 (PAI-1) quantified by RT-qPCR from the control PBS group (white bar), BLM group (pink bar) and BLM-AE group (pink hatched black bar). Effect of BLM or BLM-AE was presented as fold induction normalized to the mean value of PBS group and reported to 1. All values are represented as median ± interquartile range; one-way ANOVA analysis followed by Newman–Keuls was performed. ns: not significant, * *p* ≤ 0.01.

Immunostaining of HIF-1α protein (Figure 7A) did not evidence any increase in the lung expression of HIF-1α in the BLM group as compared to the PBS group. Of note, exacerbation induced a strong increase in the expression of HIF-1α protein, particularly in the cytoplasm of AEC (BLM-AE, Figure 7A). Moreover, mRNA expression levels of BNIP3 and SERPINE1 genes (encoding PAI-1) (two target genes of HIF), were strongly enhanced in the BLM-AE group (Figure 7B,C).

## 4. Discussion

The present study was designed to set up and characterize a new non-infectious mouse model to further study the mechanism implicated in the acute exacerbation of pulmonary fibrosis and propose new drug treatment during AE. In mice harboring a chronic and moderate pulmonary fibrosis caused by 4 IT instillations of low-dose (0.8 UI/g) BLM every 2 weeks, AE was induced by two additional instillations of a double-dose of BLM (1.6 UI/g). The last two instillations of double-dose BLM induced in one month a clear clinical aggravation with weight loss and a mortality of 40%, as well as rapid respiratory deterioration as assessed by decreased oxygen saturation and lung compliance. At the histopathological and molecular levels, exacerbation was associated with features of subacute alveolar damage superadded to chronic lung remodeling reminiscent of the UIP pattern, with massive infiltration of lung tissues by macrophages and with increased levels of pro-inflammatory cytokines in lung extracts. Finally, double-dose instillations of BLM induced alveolar expression of HIF-1a protein and of its target genes BNIP3 and SERPINE1 (PAI-1).

Acute exacerbation (AE) is a dramatic event in the natural history of IPF associated with the 80% mortality of patients within one year. IPF-AE is defined as an acute, clinically significant, respiratory deterioration of unidentifiable cause (3). It is not clear whether IPF-AE represents an intrinsic acceleration of the fibrotic lung process, or whether it is induced by external occult insults such as viral infections, micro-aspiration, or thoracic surgery on a predisposed lung. It is noteworthy that exposure to ozone and nitrogen dioxide, which induced lung oxidative stress (as bleomycin), has been correlated to an increased risk of AE of IPF, worsening overall mortality [19,20]. There is currently no efficient medical treatment for IPF-AE [21]. Therefore, novel therapeutic strategies should be developed, and the use of a suitable animal model could be most helpful. Over the past 10 years, several studies using the classical mouse model of lung fibrosis induced by administration of a single dose of BLM have been carried out to mimic IPF-AE. This model of lung fibrosis is probably not the best model to reproduce the natural progression of IPF. Indeed, lung fibrosis models developed in 14 days after a single instillation of a high dose of BLM present many disadvantages. First, they are more representative of an acute damage leading to tissue fibrosis than of a progressive fibrosing lung disease. Second, as fibrosis is limited in time and can resolve spontaneously, these models contrast to the known natural chronic and progressive evolution of human IPF (6) [22].

Based on the presumed triggers of IPF-AE, AE in murine models of BLM-induced lung fibrosis were induced by one IT instillation of either LPS [23] or gamma herpes simplex virus [12,13,14]. In all cases, the authors reported the worsening of fibrosis and increased inflammatory response, with an increase in TH17 response and increased levels of pro-inflammatory cytokines. However, although the occurrence of AE after viral infection is well-described, such virus-induced models might not be suitable for studying the molecular mechanisms associated with either “idiopathic” AE, or AE induced by non-infectious triggers. 

In the present study, we used an experimental protocol adapted from Degryse et al. and Redente et al. [10,11], consisting of six repetitive IT instillations of low-dose BLM to induce a chronic and moderate lung fibrosis in mice. According to Redente, our model presents at day 90 a stable pulmonary fibrosis with a more uniform distribution of fibrotic lesions and no reversibility of the lesions observed during the time points of the experiments. The fibrosis is attested by a large amount of soluble collagen in lung extracts and a decrease in the distensibility of the lung, i.e., lung compliance. The lungs were severely damaged and the pulmonary architecture altered. The remodeled areas covering a large fraction of the total surface of the lung were mainly extended subpleural fibrosis with intraparenchymal fibrotic masses extension. These fibrotic areas show cystic cavities (some of them reminiscent of honeycombing), alveolar collapse and airways mimicking traction bronchiectasis, as observed in the UIP pattern characteristic of IPF [24]. Immunostaining experiments revealed the presence of macrophages, T and B cell infiltrates. Noteworthy is that the pro-inflammatory cytokine levels in lung extracts from the BLM group were comparable to those in the PBS group, excluding major inflammation. In addition, as described in biopsies from IPF patients [25,26], TUNEL-positive apoptotic cells were seen (Supplemental Figure S1). We also observed the induction of cell cycle regulators p21WAF1 and p16INK4 (Supplemental Figure S2) implicated in cellular senescence and aging and mainly reported in IPF lung biopsies [27,28,29]. All together, these results indicate that repetitive instillations of low-dose BLM induce chronic and moderate lung fibrotic disease that recapitulates most of the characteristics of IPF [30].

In this chronic pulmonary fibrosis model, AE was produced by doubling the dose of BLM (1.6 instead of 0.8 UI/g) for the last two instillations in the last 30 days of the experiment (BLM-AE group) which induced a strong oxidative injury. Based on previous reports in rats [31] or mice [15], we anticipated that the oxidative stress generated by a higher dose of BLM would promote an inflammatory environment in the distal lung, which is characteristic of AE. This procedure has been chosen to get as close as possible to the clinical description of AE in IPF patients, where a degradation of lung function is observed typically within less than a month [3]. Indeed, we observed a 40% mortality as seen in patients with IPF where 50% in-hospital mortality was observed. As compared to the BLM group without exacerbation, histopathology showed superadded inflammatory and loose collagenous deposits suggesting organizing pneumonitis features as observed in the autopsies of patients deceased from IPF-AE [5]. We also observed a more extensive pulmonary remodeling with an increase in collagen content leading to a decrease in pulmonary compliance. A major observation made in the BLM-AE group was an activated inflammatory response, characterized by an increase in KC, IL1-*β* IL-6 and TNF-α pro-inflammatory cytokines in lung extracts, associated with a higher recruitment of macrophages in injured lung areas. Our cytokine data on lung extracts are in line with observations made in IPF patients with AE showing increased levels of IL-6 and IL-8 either in the bronchoalveolar lavage fluid (BALF) [32,33] or in blood [34]. Further experiments in our mice model of AE could be realized to investigate BALF or blood sample content. Finally, neither an increase in TUNEL positive cells and caspase 3/7 activities (Supplemental Figure S1) nor an induction of cell cycle regulators p21WAF1 and p16INK4 (Supplemental Figure S2) were observed in BLM-AE as compared to BLM group.

Acute exacerbations in IPF patients often lead to episodes of severe hypoxemia, as it is the case for ALI or acute respiratory distress syndrome [32]. Accordingly, in the present study, we observed in the BLM-AE group a decrease in SpO2. Although we did not directly measure oxygen tension in lung tissue, this blood desaturation strongly suggests that remodeled and inflammatory lung tissues are hypoxic. In line with this hypothesis, we observed a strong increase in HIF-1a labeling in the distal lung from BLM-AE mice as compared to BLM mice. We also found increased mRNA expression of Bnip3 and Serpin1 HIF-dependent genes, both strongly implicated in either apoptosis or autophagy [35] and in the fibrosing process [36]. Interestingly, acute exposure of AECs to environmental hypoxia favors the secretion of pro-inflammatory cytokines and/or pro-fibrotic mediators in the lung by recruitment of macrophages [37,38], and hypoxia has been proposed to participate in AEC deregulation in the pathogenic process of IPF [39,40,41,42]. Furthermore, HIF-1α has been suggested to play a critical role in regulating the inflammatory response as a decrease in the secretion of pro-inflammatory cytokines has been observed in HIF -/- mice [43]. Therefore, these results strengthen a pathogenic role of HIF-1α expression in our model of AE. 

## 5. Conclusions

In conclusion, the present study confirms that repetitive intratracheal instillations of BLM at low dose induce a chronic and stable pulmonary fibrosis in mice reproducing many characteristics of IPF. It also demonstrates that once lung fibrosis is well-established, instillation of higher doses of BLM induces a rapid clinical deterioration and histological remodeling together with acute exacerbation of pre-existing pulmonary fibrosis. This new mouse model of non-infectious exacerbation of pulmonary fibrosis is easily applicable and could represent a suitable preclinical model to test the impact of new therapeutic molecules for the treatment of IPF-AE. Futures studies are now necessary to test the relevance of the nintedanib or pirfenidone therapeutic molecules currently used in clinics to better understand their impact on AE [44].

## Figures and Tables

**Table 1:** Antibodies used for immunohistochemistry.

Antibody	References	Host	Antibody type	Dilution
F4-80	D2S9R, Cell Signaling Technology	Rabbit	Monoclonal	1:250
CD3	D4V8L, Cell Signaling Technology	Rabbit	Monoclonal	1:200
CD19	D4V4L, Cell Signaling Technology	Rabbit	Monoclonal	1:800
HIF-1α	NB100-479, Novus Biological	Rabbit	Polyclonal	1:750

**Table 2:** Primers used for real-time polymerase chain reaction.

Gene	Forward Primer	Reverse Primer
*BNIP3*	5′-TTT-GGG-ATC-TAC-ATT-GGA-AGG-C-3′	5′-GTG-CAG-ACA-CCC-AAG-GAT-CA-3′
*SERPINE1*	5′-GCA-CAA-CCC-GAC-AGA-GAC-AA-3′	5′-ATG-AAG-GCG-TCT-CTT-CCC-AC-3′
*18S*	5′-GTA-AGT-GCG-GGC-CAT-AAG-CTT-3′	5′-AGT-CAA-GTT-CGA-CCG-TCT-TCT-CA-3′

## Data Availability

Not applicable.

## Supplementary Materials

The following supporting information can be downloaded at https://www.mdpi.com/article/10.3390/cells11213379/s1, Figure S1: Measurement of apoptosis; Figure S2: Analysis of senescence markers.
