# Supplementary material for: A New Model of Acute Exacerbation of Experimental Pulmonary Fibrosis in Mice
Source: Cells. 2022 Oct 26;11(21):3379. doi: 10.3390/cells11213379 (PMC9654438; doi:10.3390/cells11213379)
Supplement: Supplementary file 1 [file cells-11-03379-s001.zip › cells-1921607-supplementary.pdf]

cells

MDPI

Supplemental Data

Evolution of lung stress markers

The stress markers classically associated with pulmonary fibrosis *i.e.* apoptosis, and the presence of senescent cells were analyzed, and the impact of AE on theses markers was investigated. No TUNEL positive cells were detected in the PBS group (Figure S1B), while numerous TUNEL positive cells were observed in the BLM group (Figure S1E). No evident difference was observed between the BLM (Figure S1E) and the BLM-AE groups (Figure S1H). Such an effect was also observed through the cleavage activity of the pro-apoptotic caspase 3/7 (Figure S1J). Caspase 3/7 activities were significantly induced in the two BLM groups (BLM, BLM-AE) as compared to the PBS group, with no significant difference between BLM and BLM-AE groups (Figure S1J).

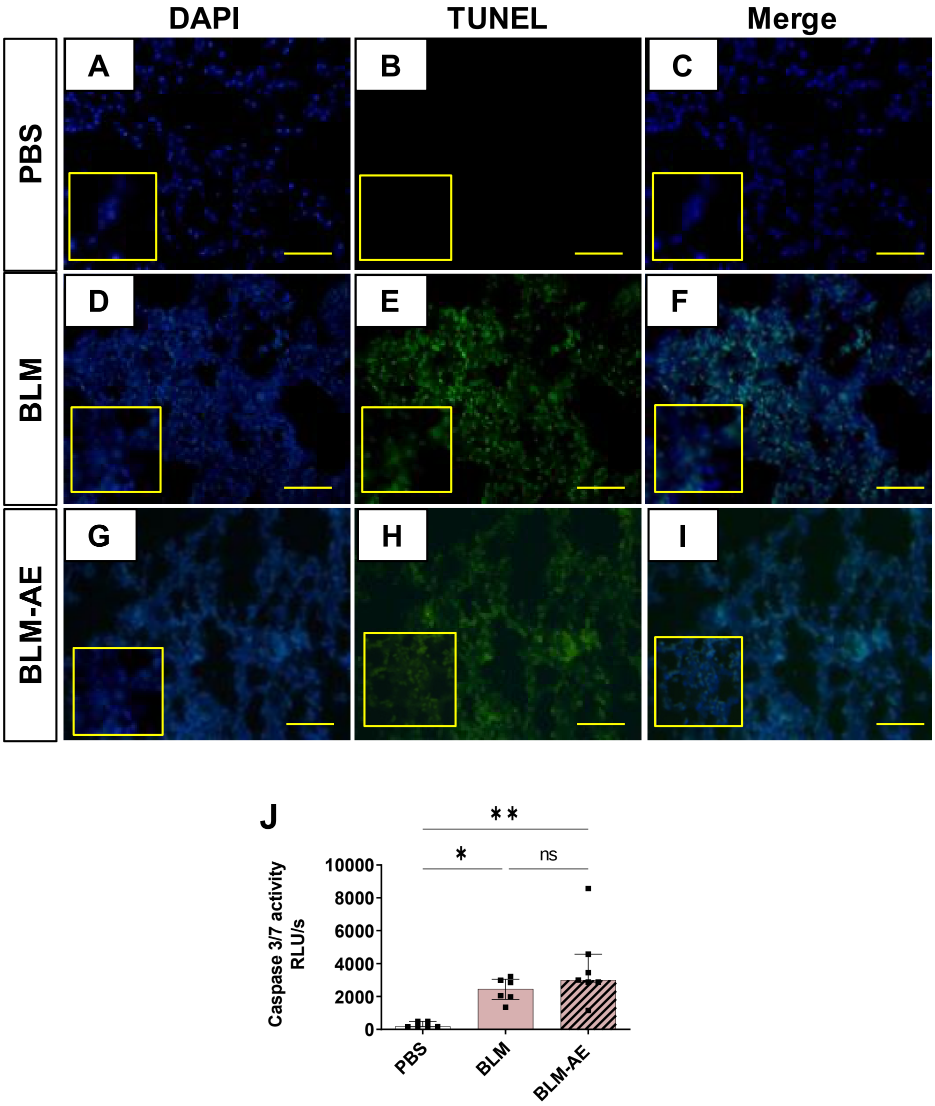

Figure S1: Measurement of apoptosis. (A-I). Evaluation of DNA breaks by the TUNEL technique. A representative image of mice from the PBS **(A, B, C),** BLM **(D, E, F)** and BLM-AE **(G, H, I)** group is shown. **(A, D, G),** Nuclei were

labeled with DAPI (blue), **(B, E, H)** 3'OH extremities were labeled with a TUNEL probe (green) and **(C, F, I)** merge was performed by Image J. Scale bar corresponding to 50µm. **(J)** Measurement of caspase 3 & 7 activities by CaspaseGlo® technique the control PBS group (white bar), BLM group (pink bar) and BLM-AE group (pink hatched black bar). All values are represented as median ± interquartile range; One-way ANOVA analysis was performed followed by Newman-Keuls test. **p≤ 0.05.

Expression of two markers associated with cell senescence ($p16^{INK4}$ protein, Figure S2A, C, E, and $p21^{WAF1}$ protein, Figure S2B, D, F) was also evaluated by immunostaining. In the BLM group, an increased staining in $p16^{INK4}$ and $p21^{WAF1}$ was observed in the fibrosis area (Figure S2C and S2D respectively) wich was more important in the BLM-AE group (Figure S2E and S2F).

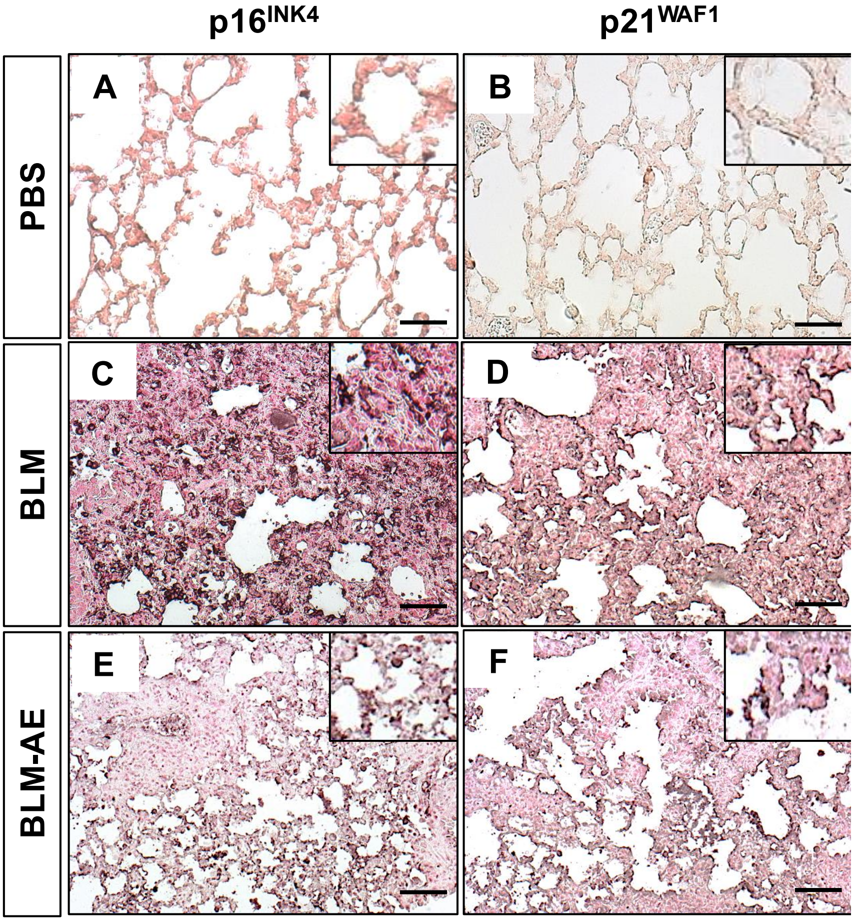

Figure S2: Analysis of senescence markers. Photographs of 5-µm serial lung sections immunostained for $p16^{INK4}$ (A, C, E) and $p21^{WAF1}$ (B, D, F) protein (scale bar corresponding to 100µm). Representatives image of PBS (n=6, A, B), BLM (n=6, C, D), BLM-AE (n=7, E, F) groups is shown. Increased expression of $p16^{INK4}$ in the BLM (C) and more in the BLM-AE (E) group is showed, mainly localized in alveolar epithelial cells in or in the vincity of fibrotic area. $p21^{WAF1}$ expression is observed only in the BLM-AE group (F), in remained tissue specifically in alveolar epithelial cells.

Materials and methods:

TUNEL staining: For detection and quantification of apoptosis, TUNEL staining in situ cell death detection kit (Roche, number #11684795910) was used following the manufacturer's recommendations on 6 mice from the PBS group, 6 mice from the BLM group and 7 mice from the BLM-AE groups. Briefly, after dewaxing, tissues were rehydrated and permeabilized with proteinase K (20ug/ml). A positive control was done using 100U DNase I. The labeling protocol was done according to the manufacturer's instructions. Slides were mounted with Vectashield Antifade mounting medium with DAPI (Vector number H-1200, UK).

Caspase 3/7 activities. Their evaluation on 6 mice from the PBS group, 6 mice from the BLM group and 6 mice from the BLM-AE groups was done by addition of a luminogenic caspase-3/7 substrate (Caspase-Glo 3/7, Promega) to total lung extract, which is cleaved in apoptotic cells to produce a luminescent signal. The assay was performed using 5µg of total protein extract. Luminescence was measured with a plate reader (Bio-TEK®).

$p21^{Waf1}$ and $p16^{Ink4}$ Immunohistochemistry: IHC was performed on tissue sections from the PBS (n=6), 6 BLM (n=6) and BLM-AE (n=7) groups. Antigen retrieval was performed in boiling citrate buffer (10mM Sodium Citrate, 0.05% Tween 20, pH 6.0). Endogenous peroxidases were quenched with 3% hydrogen peroxide for 10 minutes and sections were incubated with 5% of normal horse serum for 1 hour to block nonspecific antibody binding sites. Sections were incubated with the different primary antibodies overnight at 4° C ($p21^{Waf1}$: sc-1661, $1/500^{e}$ Santacruz; $p16^{Ink4}$, sc-397, $1/50^{e}$, Santacruz). The next day, slides were incubated with biotin-conjugated secondary antibodies (Dako REAL ™ Detection System, Peroxidase / DAB^{+}, Rabbit / Mouse) for 10 minutes and then with peroxidase-bound streptavidin (HRP) for 10 minutes. DAB (3,3'-diaminobenzidine) solution and nuclear fast red (Sigma Aldrich, ref 60700) staining were used to visualize the positive reactions.
